# Epstein–Barr virus renders the infected natural killer cell line, NKL resistant to doxorubicin-induced apoptosis

**DOI:** 10.1038/sj.bjc.6604764

**Published:** 2008-11-04

**Authors:** Y Isobe, K Sugimoto, I Matsuura, K Takada, K Oshimi

**Affiliations:** 1 Department of Hematology, Juntendo University School of Medicine, 2-1-1 Hongo, Bunkyo-ku, Tokyo 113-8421, Japan; 2 Department of Tumor Virology, Institute for Genetic Medicine, Hokkaido University, N15 W7 Kita-ku, Sapporo 060-8638, Japan

**Keywords:** natural killer-cell malignancies, Epstein–Barr virus, apoptosis, doxorubicin, nuclear factor-*κ* B

## Abstract

We established two Epstein–Barr virus (EBV)-infected NKL sublines, which acquired stress resistant phenotype against DNA damage and starvation compared with EBV-negative NKL. EBV-rendered doxorubicin resistance at least partially through NF-*κ*B activation and the resultant sustenance of antiapoptotic proteins including Bcl-X_L_ and FLIP_L/S_.

Natural killer (NK)-cell malignancies including nasal-type NK-cell lymphoma and aggressive NK-cell leukaemia have been recently recognised as new disease entities, which frequently arise in east Asia and central America and present poor prognosis (Oshimi, 2007). These diseases are closely associated with Epstein–Barr virus (EBV) infection (Kawa-Ha *et al*, 1989; Kaneko *et al*, 1995; Oshimi, 2007). EBV genome was detected in approximately 98% of nasal-type NK-cell lymphoma cases and 83% of aggressive NK-cell leukaemia cases in Japan (Oshimi *et al*, 2002). The presence of monoclonal EBV in neoplastic NK cells indicates that EBV infection occurs prior to the clonal expansion.

EBV immortalises B cells *in vitro*. In this situation, EBV expresses several latent gene products including six EBV-determined nuclear antigens, three latent membrane proteins, and two EBV-encoded RNAs (EBERs) (Young and Rickinson, 2004). Although these EBV-derived molecules function as transactivators and signal transducers in the infected cells, most of these molecules except for EBNA1, EBERs and occasional LMP1 are not expressed in nasal-type NK-cell lymphoma (Xu *et al*, 2001). EBNA1, EBERs and LMP1 also contribute to cellular transformation by affecting cellular gene expression and protein stability (Nambo and Takada, 2002; Masucci, 2004; Young and Rickinson, 2004; Samanta *et al*, 2006). Until now, it has not sufficiently elucidated the pathogenetic role of EBV in NK-cell malignancies. Recently, we have shown that EBV directly infects human NK cells *in vitro* (Isobe *et al*, 2004). *In vitro* EBV infection of NK cells enabled us to establish two EBV-carrying NK-cell sublines and to evaluate their phenotypic changes.

## Materials and methods

### Establishment of EBV-infected NKL sublines

Cell-culture condition and infection procedure were described earlier (Isobe *et al*, 2004). We used EBV-negative NK-cell leukaemia cell line, NKL as an EBV target cell (Robertson *et al*, 1996). NKL was maintained in Iscove's modified Dulbecco's medium (Invitrogen, Carlsbad, CA, USA) containing 100 U ml^−1^ human interleukin (IL)-2 (a gift from Shionogi, Osaka, Japan). We previously confirmed that this cell line had no mutations in exon 5-to-8 of *TP53* gene (Sakajiri *et al*, 2001). Akata (EBV-positive) and BJAB (EBV-negative) were used as an EBV-producing cell and an EBV target control, respectively. Akata is infected with recombinant EBV strain containing neomycin-resistant gene (Yoshiyama *et al*, 1995). After selection with medium containing 700 *μ*g ml^−1^ of G418 (Invitrogen), EBV-infected clones were maintained for more than four years using G418-free medium. BJAB was obtained from Fujisaki Cell Center, Hayashibara biochemical laboratories Inc. (Okayama, Japan).

### Southern blot analysis, western blot analysis, and flow cytometry

Southern blot analysis was performed using EBV 1.9 kb *Xho*I probe and detected with CSPD detection system (Roche Diagnostics, Basel, Switzerland). Western blot analysis was performed using the following antibodies: anti-EBNA1 (Advanced Biotechnologies, Columbia, MD, USA); anti-EBNA2; anti-LMP1; anti-ZEBRA, (Dako, Glostrup, Denmark); anti-early antigen diffuse (EA-D), (Chemicon, Temecula, CA, USA); anti-Bcl-2 (Dako); anti-Bcl-X_L_; anti-Mcl-1 (Santa Cruz Biotechnology, Santa Cruz, CA, USA); anti-Bax (Cell Signaling Technology, Danvers, MA, USA); anti-FLIP (Alexis, Lausen, Switzerland); anti-FLIP*γ*/*δ* (Sigma-Aldrich, Stockholm, Sweden); anti-Hsp 90 (Nventa, San Diego, CA, USA); anti-p53 (Santa Cruz); anti-phosphorylated p53 (Cell Signaling Technology); and anti-*β* actin antibody (Sigma-Aldrich). Antibody signals were enhanced and detected with ECL (Amersham International plc, Buckinghamshire) or Western Blue (Promega, Madison, WI, USA). Cell-surface P-glycoprotein (P-gp) was analysed by flow cytometry using anti-P-gp antibody, MRK16 (Kyowa Medex, Tokyo, Japan).

### Cell viability and apoptosis assay

Viable and dead cell numbers were determined by trypan-blue dye exclusion. Each cell suspension (4 × 10^5^ ml^−1^) was treated with 4-hydroxycyclophosphamide (HC) (a gift from Shionogi), doxorubicin (DXR), vincristine (VCR) (Sigma-Aldrich), and I-*κ*B kinase inhibitor BMS-345541 (Merck KGaA, Darmstadt, Germany). We assessed the cell viability using 3-(4,5-dimethylthiazol-2-yl)-2,5-diphenyltetrazolium bromide (MTT) assay (Promega). The viability was calculated as percent absorbance of formazan products, that is, (OD570DXR-treated/OD570control) × 100%. We also evaluated the cell death process by FITC-conjugated annexin V-binding and 7-amino actinomycin D (AAD)-rejection assays (Beckman Coulter, Fullerton, CA, USA). DNA nick ends in apoptotic cells were labelled with fluorescein isothiocyanate (FITC)-conjugated dUTP using Mebstain Apoptosis Kit (Beckman Coulter) and analysed by flow cytometry.

### Nuclear factor-*κ*B activity assay

DNA binding activity of nuclear factor (NF)-*κ*B was evaluated using an enzyme-linked immunosorbent assay method. Nuclear extract (10 *μ*g) was isolated from DXR-treated and untreated cells and assayed using NF-*κ*B p65 Transcription Factor assay Kit (Chemicon). Relative absorbance was calculated as follows: OD450/OD650– OD450control/OD650control.

## Results

### Establishment of EBV-carrying NKL sublines

**Figure 1:**
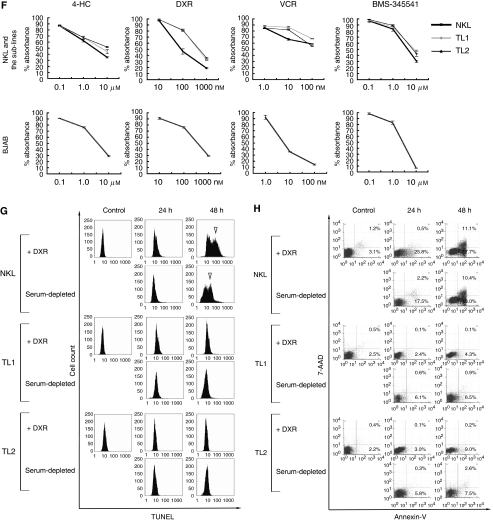
(**A**) NKL and BJAB were infected with Akata-derived EBV. Southern blot analysis detected monoclonal EBV genome in EBV-infected NKL cells. The blotting signal for clone 2 (lane 4) is weaker than those of established EBV-infected NKL sublines named TL1 (lane 5) and TL2 (lane 6), suggesting that clone 2 should contain less copy number of EBV. EBV-infected BJAB appears to consist of two clones (lane 2). (**B**) TL1 (lane 5) and TL2 (lane 6) express EBNA1 and LMP1, but lack EBNA2 and lytic marker proteins, ZEBRA and EA-D. TPA-treated Akata (lane 1) and EBV-infected BJAB (lane 2) are positive controls of lytic and latent phases, respectively. (**C**) Time courses of cell count for NKL, TL1, and TL2 at steady state. TL1 and TL2 showed no growth advantage compared with NKL. Alive (trypan-blue negative)- and dead (trypan-blue positive)-cell counts are shown in gray- and black-bars, respectively. (**D**) Time courses of cell count for NKL, TL1, and TL2 after treatment with 150 nM of doxorubicin (DXR). After 48 h, approximately 60% of NKL cells were dead, whereas above 85% of TL1 and TL2 cells survived. (**E**) Change in cell morphology after 48 h of treatment with DXR in three lines (Wright–Giemsa stain × 1000). Although both TL1 and TL2 cells had no apparent change in their cell morphology, about half of NKL cells underwent apoptosis (arrowheads). (**F**) A cell viability assay using 3-(4,5-dimethylthiazol-2-yl)-2,5-diphenyltetrazolium bromide after 48 h of treatment with 4-hydroxycyclophosphamide, DXR, vincristine, and BMS-345541, TL1 and TL2 showed resistance to DXR and VCR treatment compared with NKL. (**G**) Flow cytometric terminal deoxynucleotidyl transferase-mediated dUTP nick end-labeling (TUNEL) assay after treatment with 150 nM of DXR or serum depletion (from 10 to 0.1% of fetal bovine serum). Although about half of NKL cells underwent apoptosis after each treatment, essentially no apoptotic populations were detected in TL1 and TL2. Open arrowheads show TUNEL-positive populations. (**H**) 7-amino actinomycin D rejection and annexin V-binding assay after treatment with DXR or serum depletion. About half of NKL cells became positive for annexin V after each treatment. In contrast, approximately 90% of TL1 and TL2 cells were negative for annexin V after each treatment.

We initially obtained three G418-resistant NKL subclones containing monoclonal EBV genome (Figure 1A). During the 4 years of incubation, clone 2 stopped proliferation, and we finally established two EBV-infected sublines named TL1 and TL2. Western blot analysis showed expression of EBNA1 in all EBV-infected subclones (Figure 1B). EBNA2 and lytic marker proteins, ZEBRA and EA-D were absent in these clones. In addition, LMP1 was detected in TL1 and TL2 but not in clone 2. LMP1 in TL1 and TL2 showed smaller sizes than that in EBV-infected BJAB (Figure 1B). These truncated forms resemble lytic LMP1 detected in TPA-treated Akata (Figure 1B). Because lytic marker proteins were absent in both sublines, detection of the truncated forms may represent rapid turnover of LMP1 in TL1 and TL2.

### EBV infection of NKL showed no growth advantage but rendered the infected cells resistant to doxorubicin-induced apoptosis

NKL and EBV-infected TL1 and TL2 showed no difference in cell growth at a steady state (Figure 1C). After treatment with 150 nM of DXR, both sublines remained more viable than NKL, that is, although approximately 60% of NKL cells became positive for trypan-blue dye exclusion assay after 48 h, less than 14% of TL1 and TL2 cells were dead (Figure 1D). Indeed, about half of NKL cells underwent apoptosis after the DXR treatment (Figure 1E). We also evaluated possible difference of drug sensitivity among NKL, TL1 and TL2 using 4-HC, DXR, VCR, and I-*κ*B kinase inhibitor BMS-345541. After 48 h of treatment, MTT assay showed that TL1 and TL2 were more resistant to DXR than NKL (Figure 1F). Although NKL itself was rather resistant to VCR compared with BJAB, the sublines were a little more refractory to the agent. The effect of 4-HC or BMS-345541 on cell viability was almost equal between NKL and its sublines. Flow cytometric terminal deoxynucleotidyl transferase-mediated dUTP nick end-labelling assay showed that treatment with 150 nM of DXR or serum depletion (10% to 0.1%) for 48 h induced apoptosis in about half of NKL cells but in only few TL1 and TL2 cells (Figure 1G). Annexin V-binding and 7-AAD-rejection assays confirmed the loss of susceptibility to DXR and serum depletion in TL-1 and TL-2 (Figure 1H). These results showed that EBV rendered the infected NKL cells resistant to various cell stresses like DNA damage and starvation.

**Figure 2:**
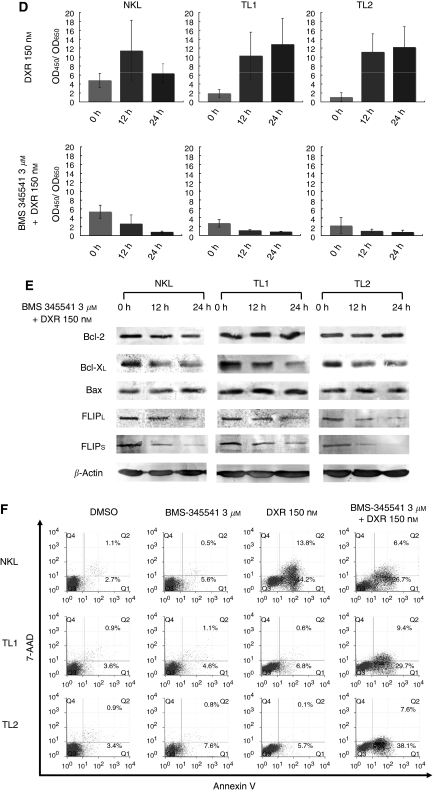
(**A**) Western blot analysis showed essentially the same expression levels of Bcl-2, Bcl-X_L_, Mcl-1, Bax, FLIP_L/S_, and p53 among NKL, TL1 and TL2. (**B**) Cell-surface P-glycoprotein was absent not only in NKL but also TL1 and TL2 by flow cytometry. Open histograms show negative control with isotype-matched control antibody. (**C**) Alterations of antiapoptotic protein levels in NKL and two sublines after doxorubicin (DXR) treatment. Although NKL declined expression levels of Bcl-2, Bcl-X_L_, and FLIP_L/S_ after 24 h of DXR treatment (left column), TL1 (middle column) and TL2 (right column) kept to express these proteins, and showed rather increased expression levels of Bcl-X_L_ and FLIP_L/S_. (**D**) NF-*κ*B (p65 subunit) binding activity after DXR treatment. Although the binding activity in NKL remained to be its basal level at 24 h, those in TL1 and TL2 increased approximately five times after 12 h and further boosted at 24 h. In contrast, NF-*κ*B inhibitor BMS-345541 almost completely suppressed DXR-induced NF-*κ*B activation in all three lines. Each experiment was performed in triplicate. Each error bar represents s.d. (**E**) Alterations of antiapoptotic protein levels in NKL and two sublines after treatment with both DXR and BMS-345541. In the presence of BMS-345541, treatment with DXR clearly decreased expression levels of Bcl-X_L_ and FLIP_L/S_ even in TL1 and TL2. Expression level of Bcl-2 was decreased only in NKL. (**F**) 7-amino actinomycin D rejection and annexin V-binding assay after treatment with DMSO (control), BMS-345541, DXR, and both BMS-345541 and DXR. Although approximately 5–7% of apoptotic populations were detected in TL1 and TL2 after treatment with 3 *μ*M of BMS-345541 and 150 nM of DXR, respectively, those mixed treatments induced apoptosis in approximately 40% of populations in TL1 and TL2.

We next evaluated possible alterations of antiapoptotic proteins after EBV infection of NKL cells. Western blot analysis showed essentially the same levels of Bcl-2, Bcl-X_L_, Mcl-1, Bax, p53 and FLIP_L/S_ among NKL, TL1 and TL2 (Figure 2A). Expression of *MDR1*-encoded P-gp was absent in all these lines, which confirmed that the resistance to DXR should be unrelated to drug pumping (Figure 2B). Although no apparent difference was detected at the steady state, treatment with 150 nM of DXR for 24 h reduced expression levels of Bcl-2, Bcl-X_L_, and FLIP_L/S_ specifically in NKL (Figure 2C). The amounts of these proteins were constant or rather increased in TL1 and TL2 after the same treatment. Protein levels of Mcl-1 and Bax were unchanged in all three lines (Figure 2C). Increased levels of p53 phosphorylation at serine-15 after DXR treatment indicated preservation of normal p53 activity in all three lines (Figure 2C). Because nuclear factor (NF)-*κ*B is reported to affect expression levels of Bcl-X_L_ and FLIP_L/S_ (Karin, 2006), we examined possible involvement of NF-*κ*B in the EBV-mediated resistance to DXR. In spite of a transient increase at 12 h, target sequences-binding activity of NF-*κ*B p65 clearly regressed after 24 h of DXR treatment in NKL (Figure 2D). In contrast, although the basal activities in TL1 and TL2 were rather suppressed, they increased approximately five times as high as their basal levels after 12 h of DXR treatment, and were further boosted at 24 h (Figure 2D). However, NF-*κ*B inhibitor BMS-345541 almost completely suppressed DXR-induced NF-*κ*B activation in all three lines. In the presence of this inhibitor, treatment with DXR clearly decreased expression levels of Bcl-X_L_ and FLIP_L/S_ and induced massive apoptosis even in TL1 and TL2 (Figure 2E and F). It should be noted that BMS-345541 rather decreased the apoptotic cell percentage from approximately 60% to around 35% in DXR-treated NKL. These experiments were repeated at least three times with essentially the same results.

## Discussion

In this study, we established TL1 and TL2, which contain monoclonal EBV genome and showed type II latency. Their manner of infection corresponds to the clinical setting of NK-cell malignancies (Xu *et al*, 2001). The intense signals in Southern blot analysis suggested that TL1 and TL2 should contain greater copy number of EBV genome than clone 2 (Figure 1A). Relatively high copy number of EBV may contribute to successful maintenance of TL1 and TL2 over 4 years. We previously detected truncated forms of LMP1 at a later phase of latent infection (Isobe *et al*, 2004). Although full-length LMP1 induces NF-*κ*B activation, its truncated form is reported to inhibit this effect (Erickson and Martin, 2000). The canonical function of LMP1 seems to be limited and might not fully contribute to establish the latent infection in both NKL sublines. These truncated forms might explain the relatively low basal NF-*κ*B activity in TL1 and TL2 compared with NKL. Of course, latent EBV infection may exert a supportive role for the NF-*κ*B pathway and might lessen its activity without altering basal cell growth activity in both sublines.

At a steady state, TL1 and TL2 had no growth advantage and showed essentially the same expression levels of several antiapoptotic proteins as NKL. However, these EBV-infected sublines were apparently more resistant to DXR and serum depletion than NKL. The results indicate that EBV infection conferred resistance to various cell stresses like DNA damage and starvation in NKL. Expression levels of Bcl-2, Bcl-X_L_ and FLIP_L/S_ decreased in NKL but not in two sublines after DXR treatment. In the presence of NF-*κ*B inhibitor BMS-345541, both NKL and the EBV-infected sublines were almost equally sensitive to DXR and failed to maintain the expression levels of Bcl-X_L_ and FLIP_L/S_ after DXR treatment. The results are completely in line with the previous report that constitutively activated NF-*κ*B has shown to maintain high expression levels of Bcl-X_L_ and FLIP_L/S_ and confer resistance to some anticancer drugs (Wang *et al*, 1999; Karin, 2006). EBV-associated gene products like EBNA1 and LMP1 were reported to modify the ubiquitin–proteasome activity and affect proteolysis of various cellular proteins including NF-*κ*B (Masucci, 2004). In addition, EBERs appear to be involved in modulation of cellular response and were reported to activate NF-*κ*B-signalling pathway (Nambo and Takada, 2002; Samanta *et al*, 2006). These observations strongly argue that the presence of EBV must render NKL more resistant to lethal stress like DNA damage at least partially through the NF-*κ*B pathway.

During a series of experiments aimed to show the contribution of NF-*κ*B activity in stress resistance of TL1 and TL2, we found that BMS-345541 reproducibly attenuated DXR-induced apoptosis in NKL cells. Atypical activators of NF-*κ*B including DXR have been shown to convert p65 from a transcriptional activator into a transcriptional repressor of antiapoptotic genes including Bcl-X_L_ and FLIP_L/S_ (Campbell *et al*, 2004; Dutta *et al*, 2006). We believe that DXR may induce apoptosis in NKL at least partially through this atypical activation of NF-*κ*B. Prolonged activation of NF-*κ*B during DXR treatment maintained high expression levels of Bcl-X_L_ and FLIP_L/S_ in the EBV-infected sublines. Therefore, we suppose that in the presence of some EBV-associated gene products, even DXR treatment may preferentially elicit classical NF-*κ*B activity with an antiapoptotic feature rather than inducing the pro-apoptotic situation and thus render the cell resistant to apoptosis. Abrogation of both positive and negative impacts of NF-*κ*B on apoptosis by BMS-345541 should bring about essentially the same behaviour of NKL, TL1 and TL2 toward the DXR treatment.

In conclusion, we report here *in vitro* establishment of two EBV-infected NKL sublines, TL1 and TL2. Although the infected sublines had no growth advantage compared with NKL, they showed a resistant phenotype to stress-induced apoptosis at least partially through the NF-*κ*B activation and the resultant sustenance of Bcl-X_L_ and FLIP_L/S_. We believe that this model suggests the contribution of EBV in NK-cell tumorigenesis.
